# The Prognostic Value of the Combination of Low VEGFR-1 and High VEGFR-2 Expression in Endothelial Cells of Colorectal Cancer

**DOI:** 10.3390/ijms19113536

**Published:** 2018-11-09

**Authors:** Nicky D’Haene, Caroline Koopmansch, Yves-Rémi Van Eycke, Françoise Hulet, Justine Allard, Sarah Bouri, Sandrine Rorive, Myriam Remmelink, Christine Decaestecker, Calliope Maris, Isabelle Salmon

**Affiliations:** 1 Department of Pathology, Erasme Hospital, Université Libre de Bruxelles (ULB),1070 Brussels, Belgium; Caroline.koopmansch@erasme.ulb.ac.be (C.K.); sarah.bouri@erasme.ulb.ac.be (S.B.); sandrine.rorive@erasme.ulb.ac.be (S.R.); myriam.remmelink@erasme.ulb.ac.be (M.R.); calliope.maris@erasme.ulb.ac.be (C.M.); isabelle.salmon@erasme.ulb.ac.be (I.S.); 2 DIAPath—Center for Microscopy and Molecular Imaging, ULB, 6041 Gosselies, Belgium; yveycke@ulb.ac.be (Y.-R.V.E.); allard.justine@ulb.ac.be (J.A.); cdecaes@ulb.ac.be (C.D.); 3 Laboratory of Image, Signal Processing and Acoustics, Ecole Polytechnique de Bruxelles, ULB, 1050 Brussels, Belgium; 4 CMP-Cerba European Labs, 1070 Brussels, Belgium; fhulet@labocmp.be

**Keywords:** VEGFR-1, VEGFR-2, endothelial cells, colorectal cancer, prognosis

## Abstract

Research on tumor angiogenesis has mainly focused on the vascular endothelial growth factor (VEGF) family and on methods to block its actions. However, reports on VEGF receptor (VEGFR) expression in tumor-associated endothelial cells (ECs) are limited. Thus, we evaluated VEGF, VEGFR-1 and VEGFR-2 expression in ECs of colorectal cancer (CRC) using immunohistochemistry. VEGF, VEGFR-1 and -2 expression in ECs was quantitatively evaluated by digital image analysis in a retrospective series of 204 tumor tissue samples and related to clinical variables. The data show that the VEGF, VEGFR-1 and VEGFR-2 expression in ECs is heterogeneous. Multivariate analysis including a set of clinicopathological variables reveals that high EC VEGFR-1 expression is an independent prognostic factor for overall survival (OS). The combination of low VEGFR-1 and high VEGFR-2 expression in ECs outperforms models integrating VEGFR-1 and VEGFR-2 as separate markers. Indeed, this VEGFR-1_VEGFR-2 combination is an independent negative prognostic factor for OS (*p* = 0.012) and metastasis-free survival (*p* = 0.007). In conclusion, this work illustrates the importance of studying the distribution of VEGF members in ECs of CRC. Interestingly, our preliminary data suggest that high VEGFR-1 and low VEGFR-2 expression in ECs appear to be involved in the progression of CRC, suggesting that targeting EC VEGFR-1 could offer novel opportunities for CRC treatment. However, a prospective validation study is needed.

## 1. Introduction

Angiogenesis, which refers to the formation of new blood vessels, plays an essential role in tumor growth and metastasis to another organ [1,2]. 

Research on tumor angiogenesis has mainly focused on vascular endothelial growth factor (VEGF). VEGF is a key regulator of physiologic angiogenesis and plays a major role in the pathobiology of cancer [3]. The VEGF family includes five glycoproteins (VEGF-A, VEGF-B, VEGF-C, VEGF-D and placental growth factor (PlGF)) and three receptors (VEGF receptor (VEGFR)-1, VEGFR-2 and VEGFR-3). Among the receptors, VEGFR-2 is considered the principal transducer of VEGF-dependent angiogenic signals [2,4,5,6]. The function of VEGFR-1 is more controversial. In physiological angiogenesis, VEGFR-1 is considered a decoy receptor that down-regulates angiogenesis by sequestering a VEGF, thereby precluding VEGF binding to its receptor, VEGFR-2. In contrast, upregulation of VEGFR-1 under pathological conditions is associated with stimulation of angiogenesis [4,6]. VEGFR-3 is implicated in lymphangiogenesis in physiological and pathological conditions and only in angiogenesis during pathological conditions [7].

Antiangiogenic drugs targeting the VEGF family have been approved for the treatment of a variety of tumors [8]. Bevacizumab and aflibercept are antiangiogenic agents that have been approved for the treatment of metastatic colorectal cancer (CRC). Unfortunately, resistance and several toxicities are associated with these therapies [2]. Moreover, there is no predictive marker of response to these therapies and treatments are given without patient selection [9]. Different angiogenic factors have been explored as potential biomarkers of prognosis and of prediction of tumor response to therapy in CRC. For example, circulating VEGF-A or a PlGF and VEGF-A gene polymorphism have been investigated as prognostic or predictive biomarkers, with sometimes, controversial results [10]. The heterogeneity in the methodology could explain these differences. VEGF-A appeared to be a prognostic biomarker, but its predictive role is not proved [10]. Different factors should be considered in parallel with VEGFs, taking into account factors related to maturation and stabilization of new vessels. Indeed, different studies have shown that tumor blood vessels differ from their normal counterparts in several important ways (i.e., in structure and function and in space and time). Their properties differ across different tumor types, across blood vessels of a single tumor and across different stages of tumor progression [11,12,13].

Over the past decades, many studies have assessed the expression of the VEGF family members and their prognostic value in CRC. The literature reports several controversial results for VEGFR-1, -2 and -3 expression in CRC tumor cells. Although VEGFRs are mostly expressed in endothelial cells (ECs) [14], only one study has focused on the prognostic value of VEGFR expression in CRC endothelial cells. Jayasinghe et al. demonstrated that endothelial VEGFR-3 expression was associated with distant metastatic status in CRC [15]. To the best of our knowledge, no study has analyzed the prognostic value of VEGFR-1 and VEGFR-2 expression in ECs of CRC. 

In this study, we further characterize CRC ECs by studying the expression of these two receptors and evaluating their prognostic value. For this purpose, we quantitatively evaluated the immunohistochemical expression of the VEGF, VEGFR-1 and VEGFR-2 in ECs of a retrospective CRC series.

## 2. Results

### 2.1. Study Population

Tumor samples from 259 cases were included. For 204 of the 259 patients, 10 or more blood vessels, which were required for our quantitative analysis, were manually marked off for VEGFR1 and VEGFR2. In addition, VEGF analysis was performed for 130 patients. The clinicopathological characteristics of these patients are summarized in Table 1. The histological type for all cases was adenocarcinoma.

### 2.2. VEGF, VEGFR-1 and VEGFR-2 Expression in CRC ECs

**Figure 1:**
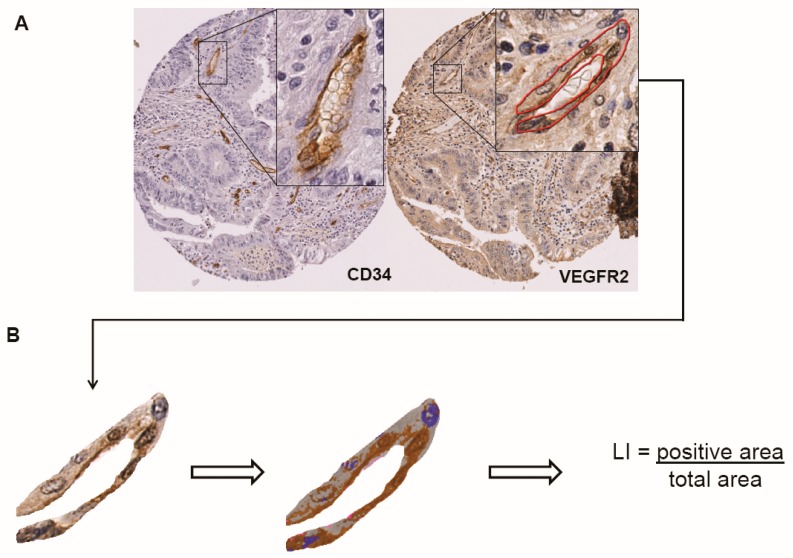
Quantitative image analysis on Tissue MicroArray (TMA): (**A**) After digitization of the TMA slides with a NanoZoomer Digital Slide Scanner (Hamamatsu, Hamamatsu City, Japan), blood vessels, highlighted by anti-CD34 immunostaining, were manually marked on the digital vascular endothelial growth factor receptor (VEGFR) slides by a pathologist using the NDP.view software. The tissue core diameter is 600 µm. (**B**) The delineated regions were imported into the Visiopharm software to quantify the VEGF, VEGFR-1 or VEGFR-2 expression levels using the labelling index (LI), i.e., the percentage of the immunoreactive area within the vessel area.

As illustrated in Figure 1, the glands and blood vessels were both labelled with the anti-VEGF and VEGFR immunostainings. Therefore, we used image analysis tools, as described in the Materials and Methods and the Figure 1 legend, to quantitatively and specifically study the VEGF, VEGFR-1 and VEGFR-2 expression in ECs.

**Figure 2:**
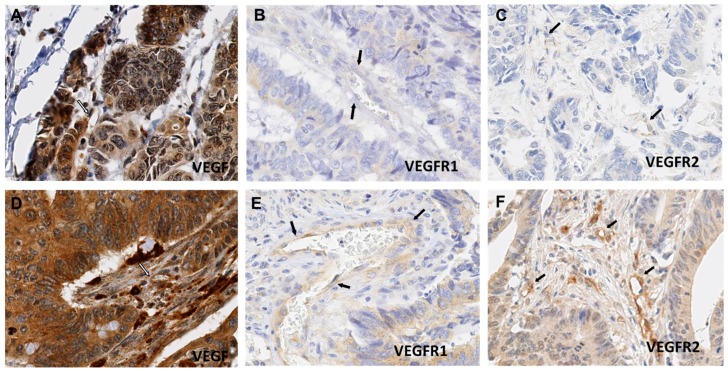
Heterogeneous VEGF, VEGFR-1 and VEGFR-2 expression in colorectal cancer (CRC) endothelial cells (ECs): VEGF expression in ECs was heterogeneous with an labelling index (LI) range from 10.9 to 90% (**A**,**D**); Anti-VEGFR-1 immunostaining shows rare positivity with an LI range from nearly 0 (**B**) to 20% (**E**) in endothelial cells. VEGFR-2 shows a much wider immunostaining, with an LI range from 10 (**C**) to 72% (**F**). (Magnification: 400×). The arrows are pointed at vessels.

VEGF expression in ECs was heterogeneous (Figure 2A,D) with a median labelling index (LI) of 45.3% (min–max: 10.9–90%). We also observed VEGFR-1 expression heterogeneity in ECs (Figure 2B,E) with a median LI of 1.8% (min–max: 0–24.1%). Similarly, VEGFR-2 expression in ECs was heterogeneous (Figure 2C,F) with a median LI of 41.1% (min–max: 8.6–72.4%). No significant correlation was found between the VEGFR-1 LI and VEGFR-2 LI (Spearman’s correlation coefficient = 0.1, *p* > 0.05).

VEGF, VEGFR-1 and VEGFR-2 expression in ECs did not vary significantly across all the clinicopathological characteristics analyzed, except for a significant association between tumor grading and VEGF expression in ECs (Table 1). Moderately and poorly differentiated CRC exhibited higher VEGF LIs than well differentiated CRC (*p* = 0.007).

### 2.3. Prognostic Evaluation of VEGF, VEGFR-1 and VEGFR-2 Expression in ECs of CRCs

**Figure 3:**
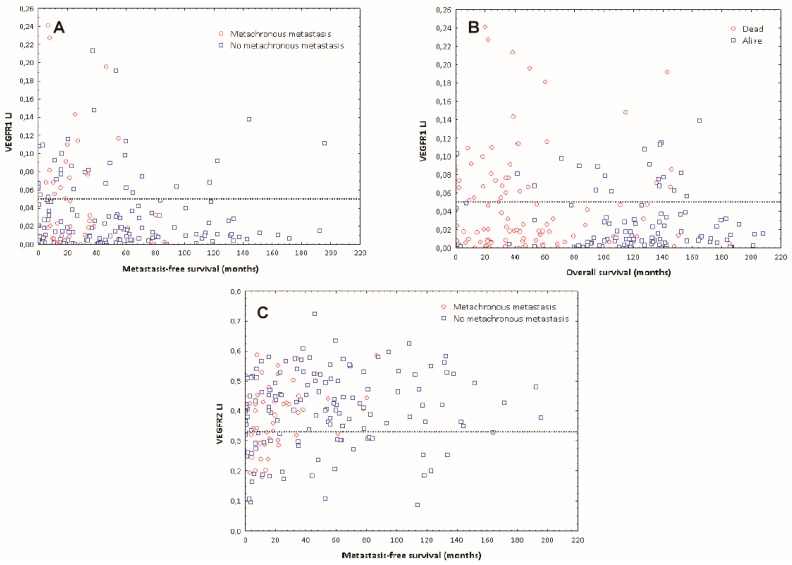
Distribution of VEGFR-1 and VEGFR-2 expression in ECs relative to survival: (**A**) LI values ≤ 5% for VEGFR-1 expression in CRC ECs identify the majority of patients with a low metastasis risk. Red/blue dots identify metastatic/metastasis-free patients, respectively. (**B**) LI values ≤ 5% for VEGFR-1 expression in CRC ECs identify the majority of patients with improved overall survival. Red/blue dots identify dead/alive patients respectively. (**C**) LI values ≥ 33% for VEGFR-2 expression in CRC ECs identify the majority of patients with a low metastasis risk. Red/blue dots identify metastatic/metastasis-free patients respectively.

Using univariate Cox survival analyses, we observed that VEGF expression in ECs of CRCs is not associated with metastasis-free survival (*p* = 0.38) or overall survival (*p* = 0.18). In contrast, low VEGFR-2 (Hazard Ratio (HR) = 0.98; *p* = 0.043) and high VEGFR-1 (HR = 1.06; *p* = 0.031) expression in ECs were associated with poor metastasis-free survival (MFS) and high VEGFR-1 expression in ECs was also associated with poor overall survival (OS) (HR = 1.06; *p* = 0.004). MFS was calculated as the time between surgical intervention and detection of metastasis on imaging; OS was calculated as the time between surgery and death, as officially registered. Based on these results, we analyzed the distribution of the VEGFR-1 and VEGFR-2 LI values. We found that the majority of patients with good OS and/or good MFS were characterized by a VEGFR-1 LI lower than 5% and/or a VEGFR-2 LI higher than 33% (Figure 3). 

**Figure 4:**
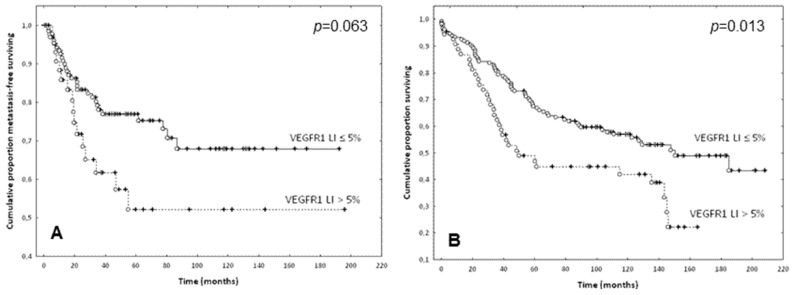

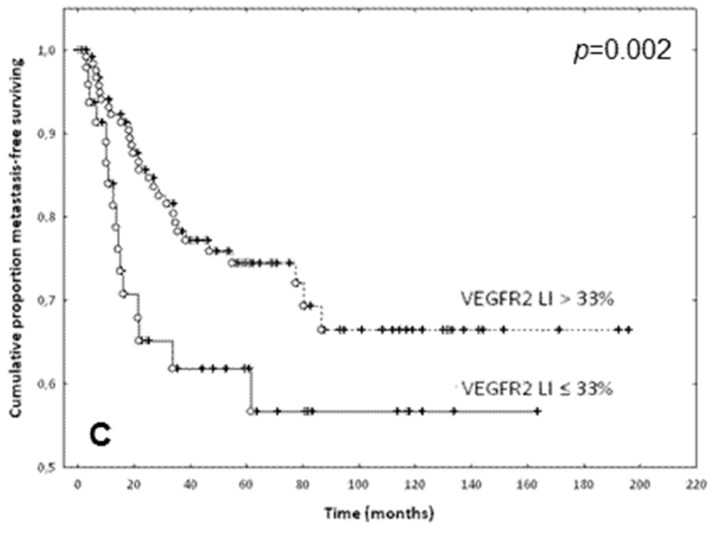
Prognostic value of VEGFR-1 and VEGFR-2 expression in EC of CRC: (**A**) Metastasis-free survival curves of patients dichotomized based on VEGFR-1 LI ≤ (solid line) or > (dotted line) 5% (*p* = 0.063). (**B**) Overall survival curves of patients dichotomized based on VEGFR-1 LI ≤ (solid line) or > (dotted line) 5% (*p* = 0.013). (**C**) Metastasis-free survival curves of patients dichotomized based on VEGFR-2 LI ≤ (solid line) or > (dotted line) 33% (*p* = 0.002). Complete and censured data are shown as dots and crosses, respectively.

The Kaplan-Meier curves and the Wilcoxon-Gehan tests confirmed the prognostic potential of these thresholds (Figure 4). 

Given these data, we hypothesized that the combination of the two conditions (i.e., VEGFR-1 LI lower than 5% and VEGFR-2 LI higher than 33%) should identify a group with a good prognosis. Thus, the study population was divided into two groups as follows:

- The first group included cases with a VEGFR-1 LI ≤ 5% and a VEGFR-2 LI > 33% (*n* = 101)
- The second group included cases with a VEGFR-1 LI > 5% and/or a VEGFR-2 LI ≤ 33% (*n* = 103).

**Figure 5:**
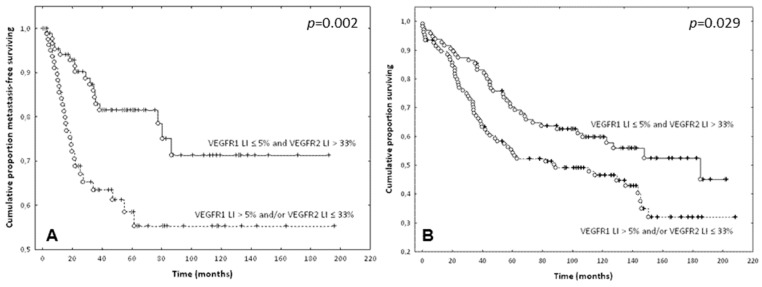
Prognostic value of the combination of the VEGFR-1 and VEGFR-2 expression in ECs of CRC: (**A**) Metastasis-free survival curves of patients dichotomized based on the combination of VEGFR-1-LI and VEGFR-2-LI (*p* = 0.002). (**B**) Overall survival curves of patients dichotomized in a similar manner (*p* = 0.029). Complete and censured data are shown as dots and crosses, respectively. Solid lines identify patients with ECs characterized by a VEGFR-1 LI ≤ 5% and a VEGFR-2 LI > 33%. Dotted lines identify patients with ECs characterized by a VEGFR-1 LI > 5% and/or a VEGFR-2 LI ≤ 33%.

The Kaplan-Meier curves and the Wilcoxon-Gehan test showed that the patients of the first group (VEGFR-1 LI ≤ 5% and VEGFR-2 LI > 33%) had longer MFS (*p* = 0.002) and OS (*p* = 0.029) (Figure 5).

Using multivariate survival analyses, we first identified the clinicopathological variables (described in Table 1) with an impact on the prognosis in the series of 204 CRC patients. The results showed that age (HR = 1.04; *p* = 0.0008), N stage (HR = 2.61; *p* < 10^−6^) and absence of postoperative treatment (HR = 0.48 for treatment administration; *p* = 0.01) were independent negative prognostic factors for OS. In contrast, only N stage (HR = 2.89; *p* < 10^−6^) was significant for MFS. By adding either VEGFR-1 LI or VEGFR-2 LI to the prognostic clinicopathological variables in the multivariate survival models, we observed that only VEGFR-1 expression was an independent prognostic factor for poor OS (HR = 1.06; *p* = 0.004, Table 2). 

Finally, we similarly tested the contribution of the abovementioned “VEGFR-1_VEGFR-2” combination and identified it as an independent prognostic factor for both MFS (HR = 2.31; *p* = 0.007) and OS (HR = 1.7; *p* = 0.012) (Table 3).

## 3. Discussion

In the present study, VEGFR-1 expression in CRC ECs is identified as a poor prognostic factor for MFS and OS in the trough univariate analysis and for OS in the trough multivariate analysis that took clinicopathological variables into account. To the best of our knowledge, this EC-related association has never been described for CRC. In prostate cancer, one study showed that strong endothelial VEGFR-1 expression appeared to be an independent predictor of distant relapse [16]. In epithelial CRC tumor cells, the prognostic impact of VEGFR-1 is controversial, because this marker is considered either a good prognostic factor [17] or a poor one [18]. This divergence has also been observed for other cancer types. High VEGFR-1 expression in epithelial tumor cells of breast cancers and non-small cell lung carcinomas is associated with short survival [19,20,21], but low VEGFR-1 expression in tumor cells of cholangiocarcinomas and diffuse large B cell lymphomas (DLBCL) is also associated with short survival [22,23]. 

The prognostic value of VEGFR-1 expression in endothelial cells needs to be related to its role in angiogenesis, which is, however, controversial. VEGFR-1 is considered a decoy receptor in physiological angiogenesis. In contrast, for pathological conditions, including cancers, upregulation of VEGFR-1 is associated with angiogenic stimulation [4,6]. Several studies have suggested that VEGFR-1 expression is associated with a deficient coverage of vessels by mural cells and increased vascular permeability, reflecting vascular immaturity [24,25,26,27]. Such vessel immaturity could promote metastasis [28], which could explain the association between VEGFR-1 expression and MFS observed in the present study.

Regarding VEGFR-2, the univariate analysis revealed that high VEGFR-2 expression in ECs was a good prognostic factor for MFS. To the best of our knowledge, this second EC-related association has not been described for CRC. In metastatic gastric/gastroesophageal junction carcinoma, high VEGFR-2 endothelial expression has been associated with a non-significant prognostic trend toward shorter progression-free survival [29]. In CRC epithelial tumor cells and tumor cells of urothelial carcinomas, high VEGFR-2 expression has been described as a good prognostic factor [18,30,31]. Contrastingly, in tumor cells of chordomas [32], squamous cell carcinomas of the lung [33], hepatocarcinomas [34], high-grade gliomas [35] and DLBCL [23], high VEGFR-2 expression has been described as a poor prognostic factor.

In our study, the finding that high VEGFR-2 expression in ECs is a good prognostic factor is disconcerting, because VEGFR-2 is reported to be the major mediator of angiogenesis and is targeted by anti-angiogenic therapies [2,4,6]. Moreover, Phase 3 trial showed that ramucirumab, a human IgG-1 monoclonal antibody that targets the extracellular domain of VEGF receptor 2, plus FOLFIRI significantly improved OS compared with the placebo plus FOLFIRI as a second-line treatment for patients with metastatic CRC [36]. However, several in vitro studies showed that addition of VEGF and hypoxia stimulated VEGFR-1 expression and decreased VEGFR-2 levels in endothelial cells [3,37,38]. In addition, in tumor cells, VEGF expression is associated with a poor prognosis, including in patients with CRC [39]. Thus, our hypothesis is that the combination of high VEGFR-1 and low VEGFR-2 levels reflects high VEGF expression. 

Our results show that the combination of VEGFR-1 and VEGFR-2 expression in ECs outperforms models integrating VEGFR-1 and VEGFR-2 as separate markers. Indeed, this combination is an independent prognostic factor (with respect to clinicopathological variables) not only for OS but also for MFS. The added value of other biomarker combinations has been described for breast cancers [40] and pancreatic cancers [41]. These preliminary data, based on a single center retrospective study have to be validated with a prospective study including a large number of cases.

In conclusion, we showed that the combination of low VEGFR-1 and high VEGFR-2 expression in ECs was a good prognostic factor. This work emphasizes the importance of specifically studying endothelial cells, which are the principal targets of antiangiogenic therapies. In vitro studies are needed to identify the respective roles of VEGFR-1 and VEGFR-2 in tumor angiogenesis and in cancer progression. In addition, other histopathological studies are necessary to evaluate the prognostic value of this combination in other cancers. This work also underlines the importance of studying biomarker combinations. Finally, this work raises the question of targeting therapies, especially for VEGFR-1. A recent study showed that a VEGFR-1-antagonistic peptide inhibited tumor growth and metastasis [42], highlighting the importance of rethinking targets of antiangiogenic therapies.

## 4. Materials and Methods 

### 4.1. Patient Data and Tissue Microarray (TMA) Design

We retrospectively analyzed tumor samples from consecutive patients who underwent surgical resection of a primary CRC at Erasme University Hospital (Brussels, Belgium) between 1990 and 2000, without occurrence of metastatic processes at the time of surgery and without preoperative treatment. Clinical (age, gender, location survival and postoperative treatment) and histopathological (grading, T and N status) data were collected for all cases. Formalin-fixed paraffin-embedded samples were used to design tissue microarray blocks using a manual microarrayer (Beecher Instruments, Sun Prairie, WI, USA), as previously described [43]. Six tumor tissue cores of 600-µm-diameter were extracted per patient. This study was approved by the local ethics committee (Erasme Hospital–P2010/320–23/11/2010).

### 4.2. Immunohistochemistry

Standard immunohistochemistry (IHC) was applied as previously described [44,45] to serial 5-μm-thick sections to display VEGF, VEGFR-1 and VEGFR-2 expression using antibodies against VEGF (Thermo Scientific, Merelbeke, Belgium, polyclonal, dilution:1:50), VEGFR-1 (Abcam, Cambridge, UK, clone Y103, dilution 1:50) and VEGFR-2 (Cell Signaling, Danvers, MA, USA, clone 55B11, dilution 1:100). An antibody against CD34 (Abcam, Cambridge, UK, clone QBEND-10, dilution 1:200) was used to identify blood vessels. For VEGF, VEGFR-1 and CD34, IHC was performed on Discovery^XT^ (Ventana Medical Systems, Roche Diagnostics, Vilvoorde, Belgium) using streptavidin-biotin-peroxidase complex kit reagents with diaminobenzidine/H_2_O_2_ as the chromogenic substrate. For VEGFR-2, IHC was performed on BOND-MAX (Leica, Wetzlar, Germany) using the Bond Polymer Refine Detection kit (Menarini, Leica, Diegem, Belgium, kit DS9800). The sections were counterstained with hematoxylin. Negative controls were carried out by replacing the primary antibody with normal serum (Dako, Glostrup, Denmark). This control allows us to show that the labelling observed is due only to binding of the secondary antibody to the primary antibody. As previously described [41], additional technical and fixative controls were carried out by staining the TMA slides with hematoxylin-eosin and an anti-vimentin antibody (Biogenex, San Ramon, CA, USA, clone V-9, dilution 1:100), respectively. The final validation stage aimed to confirm the tumor status of the specific tissue zones targeted and the immunostaining compliance. Only the cores that satisfied all of the control tests were submitted for quantification [43].

We used the NanoZoomer 2.0-HT slide scanner (Hamamatsu, Hamamatsu, Japan) for TMA slide image acquisition and the NDP.view software (Hamamatsu) to visually assess the slides and image quality. 

### 4.3. Quantitative Image Analysis

VEGF, VEGFR-1 and VEGFR-2 labelling indices were quantified on endothelial cells specifically using the Visiomorph DP software package (Visiopharm, Hoersholm, Denmark), as previously described [43]. Briefly, on each valid TMA core, all blood vessels were identified by anti-CD34 immunostaining and manually annotated using the NDP.view software (Hamamatsu, Hamamatsu, Japan) (Figure 1). For each blood vessel, we measured the analyzed (i.e., positive and negative) tissue area and the positive (i.e., stained) area using Visiomorph. To characterize each case, we pooled all selected blood vessels and computed the labelling index (LI), which is the percentage of the immunostained EC area [43]. Only cases for which 10 or more blood vessels were available were selected for further analysis.

### 4.4. Statistical Analyses

The non-parametric Mann-Whitney and Kruskal-Wallis tests were used to compare independent groups of numerical data. Survival data were analyzed using the univariate or multivariate Cox regression method. We analyzed the set of clinicopathological variables and then selected those that showed a significant contribution (characterized by a *p*-value < 0.05) in the multivariate Cox model. Then, we added the VEGFR-1 and/or VEGFR-2 LIs to the resulting clinical model to test their potential prognostic contribution to metastasis development and mortality as outcome parameters. Metastasis-free survival (MFS) was calculated as the time between surgical intervention and detection of metastasis on imaging; overall survival (OS) was calculated as the time between surgery and death, as officially registered. The prognostic impact was also illustrated by means of the standard Kaplan-Meier analysis and the Wilcoxon-Gehan test. All statistical analyses were carried out with Statistica software (StatSoft, Maisons-Alfort, France). A *p*-value < 0.05 was considered as statistically significant.

## Figures and Tables

**Table 1:** Patient characteristics and association with endothelial cell (EC) vascular endothelial growth factor (VEGF), vascular endothelial growth factor receptor -1 (VEGFR-1) and VEGFR-2 labelling indices (LI).

	VEGFR1/VEGFR2 Analysis (*n* = 204)					VEGF Analysis (*n* = 130)		
Feature	No. of Patients	VEGFR-1 LI (mean ± SD)	*p*	VEGFR-2 LI (mean ± SD)	*p*	No. of Patients	VEGF LI (mean ± SD)	*p*
**Age (med:70; min–max: 35–92)**								
<70	101	0.04 ± 0.05	0.97	0.40 ± 0.12	0.57	62	0.47 ± 0.17	0.70
>70	103	0.04 ± 0.04		0.41 ± 0.12		68	0.49 ± 0.19	
**Sex**								
Female	95	0.04 ± 0.05	0.58	0.39 ± 0.13	0.32	64	0.48 ± 0.20	0.77
Male	109	0.04 ± 0.04		0.41 ± 0.12		66	0.47 ± 0.17	
**Location**								
Caecum	18	0.04 ± 0.04	0.29 *	0.44 ± 0.13	0.08	8	0.59 ± 0.17	0.17 **
Ascending colon	33	0.04 ± 0.05		0.4 ± 0.1		17	0.43 ± 0.18	
Hepatic flexure	1	0.1		0.6		0	NA	
Transverse colon	8	0.06 ± 0.06		0.47 ± 0.07		4	0.52 ± 0.18	
Splenic flexure	7	0.02 ± 0.03		0.42 ± 0.14		5	0.48 ± 0.15	
Descending colon	17	0.05 ± 0.06		0.4 ± 0.14		10	0.37 ± 0.20	
Sigmoid colon	47	0.03 ± 0.04		0.4 ± 0.13		34	0.46 ± 0.17	
Rectosigmoid junction	14	0.03 ± 0.03		0.38 ± 0.1		9	0.47 ± 0.19	
Rectum	48	0.04 ± 0.04		0.37 ± 0.12		35	0.51 ± 0.19	
Multiple	11	0.02 ± 0.02		0.46 ± 0.12		8	0.52 ± 0.21	
Tumor grading ***								
Well differentiated	88	0.04 ± 0.05	0.66	0.4 ± 0.13	0.77	61	0.44 ± 0.19	0.007
Moderately differentiated	107	0.03 ± 0.04		0.4 ± 0.11		63	0.52 ± 0.16	
Poorly differentiated	5	0.05 ± 0.03		0.51 ± 0.07		2	0.51 ± 0.39	
**T status**								
T1	15	0.02 ± 0.03	0.32	0.36 ± 0.12	0.10	10	0.42 ± 0.11	0.47
T2	42	0.04 ± 0.05		0.44 ± 0.12		30	0.50 ± 0.17	
T3	136	0.04 ± 0.04		0.39 ± 0.13		83	0.48 ± 0.20	
T4	11	0.04 ± 0.06		0.38 ± 0.08		7	0.48 ± 0.15	
**N status**								
N0	135	0.04 ± 0.04	0.80	0.41 ± 0.13	0.36	89	0.48 ± 0.19	0.43
N1	50	0.03 ± 0.04		0.40 ± 0.10		29	0.49 ± 0.18	
N2	19	0.05 ± 0.07		0.37 ± 0.11		12	0.45 ± 0.18	
**Postoperative treatment**								
No	137	0.03 ± 0.04	0.43	0.41 ± 0.12	0.44	88	0.48 ± 0.19	0.49
Yes	62	0.04 ± 0.05		0.4 ± 0.12		40	0.46 ± 0.18	

SD = standard deviation; *p*-values result from Mann-Whitney or Kruskal-Wallis (for more than two groups) tests. * = Hepatic flexure localization was excluded for statistical analyses. ** = the 3 localizations with n ≤ 5 were excluded for statistical analyses. *** = Moderately and poorly differentiated were pooled for statistical analyses.

**Table 2:** Multivariate analysis of overall survival for VEGFR-1 LI (*n* = 198).

Model *p*-Value	Prognostic Factors	Hazard Ratio	95% CI	*p*-Value
<10^−5^	Age	1.04	1.02–1.06	0.001
	N stage	2.72	1.97–3.75	<10^−6^
	Postoperative treatment	0.45	0.25–0.80	0.007
	VEGFR-1 LI	1.06	1.02–1.10	0.004

CI = confidence interval.

**Table 3:** Multivariate analysis of metastasis-free survival and overall survival for the VEGFR-1_VEGFR-2 combination (*n* = 198).

Model *p*-Value	Prognostic Factors	Hazard Ratio	95% CI	*p*-Value
Metastasis-free survival				
<10^−5^	N stage	2.86	1.97–4.14	<10^−6^
	VEGFR-1_VEGFR-2 *	2.31	1.44–5.03	0.007
Overall survival				
<10^−5^	Age	1.04	1.02–1.06	0.001
	N stage	2.78	2.01–3.85	<10^−6^
	Postoperative treatment	0.42	0.24–0.74	0.003
	VEGFR-1_VEGFR-2 *	1.70	1.12–2.56	0.012

* VEGFR-1_VEGFR-2 = 0 when the VEGFR-1 LI ≤ 5% and the VEGFR-2 LI > 33% and VEGFR-1_VEGFR-2 = 1 in all other cases.
